# Supplementary material for: Beyond Guidelines and Reports on Bacterial Co-/Superinfections in the Context of COVID-19: Why Uniformity Matters
Source: Antibiotics (Basel). 2022 Oct 20;11(10):1446. doi: 10.3390/antibiotics11101446 (PMC9598540; doi:10.3390/antibiotics11101446)
Supplement: Supplementary file 1 [file antibiotics-11-01446-s001.zip › antibiotics-1938641-supplementary.pdf]

antibiotics

MDPI

Supplementary File

Supplementary Material 1, Methods:

-National and international guidelines on therapeutic guidance for COVID-19:

First, we conducted a Pubmed search for national and international guidelines on therapeutic guidance for COVID-19. In the absence of guidelines, national public health organizations and societies of microbiology and infectious diseases were contacted to provide their national guidelines, if available. In cases of no reply or of absence of societies, we inquired for guidelines through infectious disease specialists from the main university hospitals of the country. Whenever necessary, guidelines were translated and evaluated for specific guidance on antibiotic prescribing in COVID-19 patients.

-Search strategy on largest (*n* > 3000) meta-analyses or prospective studies reporting on bacterial co-/superinfection rates in COVID-19 patients:

Search strategies were applied in the PubMed database.

Search method: (("covid 19"[MeSH Terms]) OR "sars cov 2"[MeSH Terms]) AND ("co infection" OR "bacterial pneumonia" OR "superinfection")).

Following papers were excluded: papers including less than 3000 participants, letters and comments, case series, unrelated papers, interventional studies.

Searched for on October 10th 2022 (selection criteria: review, systematic review, meta-analysis or clinical trial): 140 results. Four meta-analyses and one prospective original paper were retained.

Supplementary Material 2 (Table S1): National and international guidelines on COVID-19 therapeutic guidance in the European Union (EU).

Country/international society	National COVID-19 treatment guideline available (yes or no)	Any guidance regarding rational antibiotic use available (yes or no)	Separate guidance exclusively regarding rational antibiotic use (yes or no)	Description of guidelines regarding antibiotic use	Number of words dedicated to antibiotic use
Austria	No	No	No	Does not apply	0
Belgium [1]	Yes	Yes	No	English version: Confirmed COVID-19 severe disease: 'carefully consider antibiotics or antifungals according to local epidemiology' Critically ill disease: ‘track secondary bacterial and opportunistic (Aspergillus) infections’	24
Bulgaria [2]	Yes	Yes	Yes	Translated: - ‘According to the recommendations of the European Center for Disease Control, the use of antibiotics and other antimicrobial agents is indicated for suspected or confirmed bacterial co-infections or secondary infections in patients with COVID-19.’ - ‘Most of the data show a relatively low rate of bacterial coinfections and secondary bacterial infections in patients with COVID-19. They are more common in hospitalized patients with a severe course of COVID-19 and/or mechanically ventilated patients.’ -‘ In the context of the COVID-19 pandemic, the use of antibiotics and other antimicrobial drugs should be reconsidered as part of the empiric strategy for the treatment of the most severe suspected or confirmed cases of COVID-19 (patients with hypoxic respiratory failure requiring mechanical ventilation) , provided their use is	2882

				regularly reevaluated. Empirical administration of antibiotics is NOT recommended in patients with mild forms of COVID-19.'<br>-'Proposed antibiotics for empiric use are tailored to the most common causative agents of HAP/VAP: MRSA, multi-resistant A. baumannii, P. aeruginosa, K. pneumoniae, etc.'<br>-'After receiving the result of the microbiological examination, the antibiotic therapy should be reassessed according to the antibiotic sensitivity of the isolates.'	
Czech Republic [3]	Yes	Yes	No	Translated:<br>-'Distinguishing a bacterial superinfection is difficult because a significant elevation of inflammatory indicators is also accompanied by COVID-19 itself.'<br>-'However, as community bacterial superinfections (or co-infections) are not frequent (up to 10%), the effort is to reduce unnecessary antibiotic therapy.<br>-'Bacterial infections associated with<br>health care occur more often and especially in patients in intensive care. Cultures are often negative. Here, too, antibiotics are apparently used too often, and antibiotic stewardship becomes more important during the pandemic.'	165
Croatia [4]	Yes	Yes	No	Translated:<br>-' In order to exclude a more serious concomitant infection, it is necessary to perform additional microbiological diagnostics(take	92

				blood cultures, urine culture, tracheal lavage, etc.), laboratory processing (hematological, biochemical) and do a radiogram or MSCT of the thoracic organs. Infection is likely if registers leukocytosis and/or a left shift in the differential blood count with increased concentration procalcitonin and very high concentrations of CRP and IL-6. If it cannot be safely turned off infection, empiric antimicrobial therapy should be started in patients with (hospital) sepsis, especially in patients who are hemodynamically unstable.'	
Cyprus [5]	Yes	Yes	No	Translated: -'Empiric antimicrobial therapy is NOT recommended for bacterial pneumonia.' -'If there is a possibility of infection or co-infection it is recommended to start empiric antimicrobial therapy. Antimicrobial regimen is recommended or may include a macrolide.' -'If antimicrobial therapy is given for possible co-infection and there is no satisfactory clinical response to the treatment, it is recommended to expand the antimicrobial spectrum based on the epidemiological data of the hospital.'	86
Denmark [6]	Yes	Yes	No	English version: -'Antibacterial agents are of no use against viral infections. however, they are useful in the treatment of suspected/verified secondary bacterial infections.	476

	-'However it is reasonable to believe that bacterial infections are most prevalent, and variable within the patient population, co-dependent on both pulmonary (e.g. bronchiectasis and COPD) and other chronic comorbidities (e.g. diabetes, treatment with immunosuppressants). As such, there is reason to believe that secondary bacterial infections are important for disease development and the patient's prognosis.'<br>-'It is therefore recommended that at any given time, at admission or during hospitalization, if a secondary bacterial infection is suspected, antibiotic treatment should be initiated. Treatment should be broad-spectrum, as the most common etiology of secondary infections in COVID-19 is yet unknown, and since secondary infections are often caused by bacteria resistant to small-spectrum antibiotics.'<br>-'The diagnostic work-up does not differ from that in other patients; suspected bacterial infection and relevant material for microbiological investigation should be secured before the start of antibiotic treatment.'<br>-'The most frequent infection is probably pneumonia and it is unknown whether the secondary bacterial pneumonias should be considered equivalent to community-acquired pneumonia or hospital-acquired pneumonia.	

				Furthermore, disease progression in case of secondary bacterial infection has been rapid.' -'Please consult the local recommendations for antibiotic treatment in an ICU-setting Treatment of Sepsis: Please consult the Surviving Sepsis campaign COVID-19 guidelines.'	
ECDC [7]	Does not apply	Yes	No	English version: -'Antibiotics are indicated for the treatment of suspected or confirmed bacterial co-infections or secondary infections in patients with COVID-19, and are not indicated for patients with mild COVID-19. However, the prevalence of bacterial co-infection and secondary bacterial infection in patients with COVID-19 seems to be relatively low. The prevalence of secondary bacterial infections is higher in patients with severe COVID-19 who are hospitalised and/or mechanically ventilated than in other patients. On the other hand, there is a need for more clarity in defining secondary bacterial infections in COVID-19 patients.' -'The antibiotic azithromycin has been postulated as having antiviral and anti-inflammatory benefits and has been studied for the treatment of COVID-19. However, multiple studies have not identified any clinical benefit.' -'Despite the low risk of bacterial infection in	603

				patients with COVID-19 (see above), antibiotic prescribing is common in COVID-19 patients…The widespread overuse of antibiotics observed among COVID-19 patients during the pandemic runs the risk of increasing antimicrobial resistance in both inpatient and outpatient settings. In the context of the COVID-19 pandemic, antimicrobial stewardship should be strengthened to ensure appropriate use of antibiotics and other antimicrobials in COVID-19 patients.'	
ESCMID [8]	Does not apply	Yes	No	English version:<br>-'What is the effect of empirical antibiotic treatment on mortality in patients with severe COVID-19 compared with no treatment? Narrative synthesis of evidence: several RCTs have not found any effect of azithromycin compared with SOC. In the absence of RCTs assessing antibiotic use in patients with COVID-19 complicated with bacterial coinfections or secondary infections, general principles of antimicrobial stewardship should be applied. Given the low rate of bacterial coinfections, only patients with clinical or radiological suspicion of an associated bacterial infection should receive empirical antibiotics when COVID-19 is diagnosed or when hospitalization is needed.'	96
Estonia [9]	Yes	Yes	No	Translated:<br>-'COVID-19 is a viral disease and the use of routine antibacterial therapy is not indicated. There must be a	164

				specific indication for starting antibacterial therapy. When pneumonia occurs in patients with COVID-19, it is mostly a viral pneumonia at the beginning of the disease, and antibacterial treatment does not prevent the development of a secondary bacterial infection.' -'The frequency of concomitant bacterial pneumonia in hospitalized patients is less than 10%, in patients requiring intensive care 10-15%.' -'Initiation of empiric antibacterial therapy is indicated in patients with a confirmed diagnosis of COVID-19 in the following cases: radiologically extensive lung involvement, purulent sputum discharge, previous glucocorticoid treatment (≥20 mg per day >7 days of prednisolone or its equivalent), a new rise in body temperature that cannot be attributed to a given viral illness, worsening of respiratory failure or hemodynamic disturbance that cannot be attributed to the given viral disease. In the laboratory, neutrophilic leukocytosis or an increase in procalcitonin indicate the presence of a bacterial infection.'	
Finland	No	No	No	Does not apply	0
France [10]	Yes	Yes	No	Translated: -'The High Council for Public Health (HCSP) specifies the recommendations relating to the prescription of antibiotics in the context of the circulation of SARS-	94

				CoV-2. In the preamble, the HCSP emphasizes the absence of indication for antibiotic therapy in a patient with a confirmed SARS-CoV-2 infection. Nevertheless, pending confirmation of the diagnosis of SARS-CoV-2 infection, the HCSP specifies the situations which may lead to the prescription of antibiotic therapy in case of doubt with bacterial pneumonitis: -on an outpatient basis -in conventional hospitalization according to the existence or not of associated comorbidities.'	
Germany [11-13]	Yes	Yes	No	Translated: -'Azithromycin should not be administered to hospitalized COVID-19 patients as antiviral therapy'. -'Prophylactic antibiotic administration in laboratory confirmed SARS-CoV-2 infection is not recommended, given that bacterial co-infections are comparatively rare at the early stage of disease.' -'Antibiotic therapy: basically at the start of treatment in the intensive care unit and at deterioration of the patient after withdrawal of at least 2 blood culture sets. Prophylactic antibiotics are not recommended.'	100
Greece [14]	Yes	Yes	No	Translated: -'Antibiotics are given only on clinical/imaging/laboratory grounds for documented or suspected bacterial pneumonia, according to the instructions of the Hellenic Society of Infections for community-	28

				acquired or nosocomial pneumonia'	
Hungary	No	No	No	Does not apply	0
Iceland	No	No	No	Does not apply	0
Italy [15]	Yes	Yes	No	Translated:<br>-'The working group recommends carefully evaluating the absence of concomitant systemic infections and eventually set up a broad spectrum preventive antibiotic therapy scheme according to clinical indications, health policies or protocols in use.'	33
Republic of Ireland [16]	Yes	No	No	Does not apply	0
Latvia [17]	Yes	Yes	No	Translated:<br>-'According to research data, about 8% of hospitalized patients are diagnosed with COVID-19 bacterial or fungal side-infection, but up to 72% are treated with broad-spectrum antibiotics. Waiting for further research data, it would probably be wise to refuse to use antimicrobials in patients with COVID-19 and reserve them for those who have appropriate radiologic findings and/or inflammatory markers that explain secondary infection, or who are critically ill.'<br>-'In patients with documented COVID-19, empiric antibiotic therapy is not usually prescribed. Despite the lack of research data, bacterial superinfection does not appear to be a frequent feature of COVID-19.<br>However, because the clinical signs of COVID-19 can be difficult to distinguish	220

				from bacteria caused by pneumonia, empiric antimicrobial treatment of pneumonia is supported in cases where the diagnosis is not clear. Empirical treatment may be warranted in patients with documented COVID-19 if there is clinical suspicion of pneumonia - for example, episodes of new fever after a period of apyrexia with new radiological changes.'<br>-'If empiric antimicrobial is initiated treatment, it is necessary to try to determine the etiological microorganism (for example, using sputum microbiological examinations, urine antigen test) and the need to continue antimicrobial therapy should be evaluated daily. In these conditions, low procalcitonin level may be useful to exclude any potential bacterial pneumonia.<br>However, elevated procalcitonin has also been described in COVID-19, especially in late disease and does not necessarily indicate a bacterial infection.'	
Liechtenstein	No	No	No	Does not apply	0
Lithuania	No	No	No	Does not apply	0
Luxemburg	No	No	No	Does not apply	0
Malta	No	No	No	Does not apply	0
Netherlands [18]	Yes	Yes	Yes	English version:<br>-'Although the evidence base on bacterial infections in COVID-19 is currently limited, available evidence supports restrictive antibiotic use from an antibiotic stewardship perspective,	367

				especially upon admission. To support restrictive antibiotic use, maximum efforts should be undertaken to obtain sputum and blood culture samples as well as pneumococcal urinary antigen testing.<br>-'We suggest to stop antibiotics in patients who started antibiotic treatment upon admission when representative cultures as well as urinary antigen tests show no signs of involvement of bacterial pathogens after 48 hours.'<br>-'For patients with secondary bacterial respiratory infection we recommend to follow other guideline recommendations on antibacterial treatment for patients with hospital-acquired and ventilator-associated pneumonia.'<br>-'An antibiotic treatment duration of five days in patients with COVID-19 and suspected bacterial respiratory infection is recommended upon improvement of signs, symptoms and inflammatory markers.'	
Norway [19]	Yes	No	No	Does not apply	0
Poland [20]	Yes	Yes	No	English version:<br>-'Treatment with antibiotics or anti-influenza drugs is not recommended in SARS-CoV-2 infection, unless it is required because of another medical condition.'<br>-'Asymptomatic or mildly symptomatic: antibiotics and anti-influenza drugs are contraindicated, unless there is a bacterial coinfection or	80

				concomitant influenza. Fully symptomatic: antibiotic therapy in case of secondary bacterial infection. Respiratory failure (cytokine storm): antibiotic therapy in case of secondary bacterial infection. ARDS: empiric antibiotic therapy is strongly advised against unless there are evident signs of secondary bacterial infection.'	
Portugal					
Slovenia	No	No	No	Does not apply	0
Slovak Republic	No	No	No	Does not apply	0
Sweden [21]	Yes	Yes	No	Translated:<br>-'A meta-analysis of a total of 6716 patients in the USA, Europe and China showed that broad-spectrum antibiotics can be considered for seriously ill patients if concomitant bacterial infection is suspected. In the case of negative cultures, antibiotics can often be prescribed even with high CRP levels. CRP rises sharply in many cases with covid-19 pneumonitis and high CRP alone is not an indication for antibiotics.'<br>-'In the same way as with other conditions, there is an increased risk of nosocomial infections with long-term ICU care. The risk of secondary infections may increase during treatment with cortisone and other immunosuppressive treatment.'<br>-'Assessment/recommendation: Concomitant bacterial infection is unusual in covid-19 early in the course, which is why antibiotic treatment is rarely indicated initially, but	172

				broad-spectrum antibiotics can be considered in severe infection where co-infection cannot be ruled out.’	
Spain [22]	Yes	Yes	No	Translated:<br>-‘Adequate response to antibiotic treatment means achieving<br>clinical stability 3-4 days after antibiotic treatment, assessed by means of the stated criteria. Unresponsive pneumonia is pneumonia with an inadequate clinical response despite antibiotic treatment.’	44

ECDC: European Centre for Disease Control and Prevention; ESCMID: European Society of Clinical Microbiology and Infectious Diseases.

Supplementary Material 3

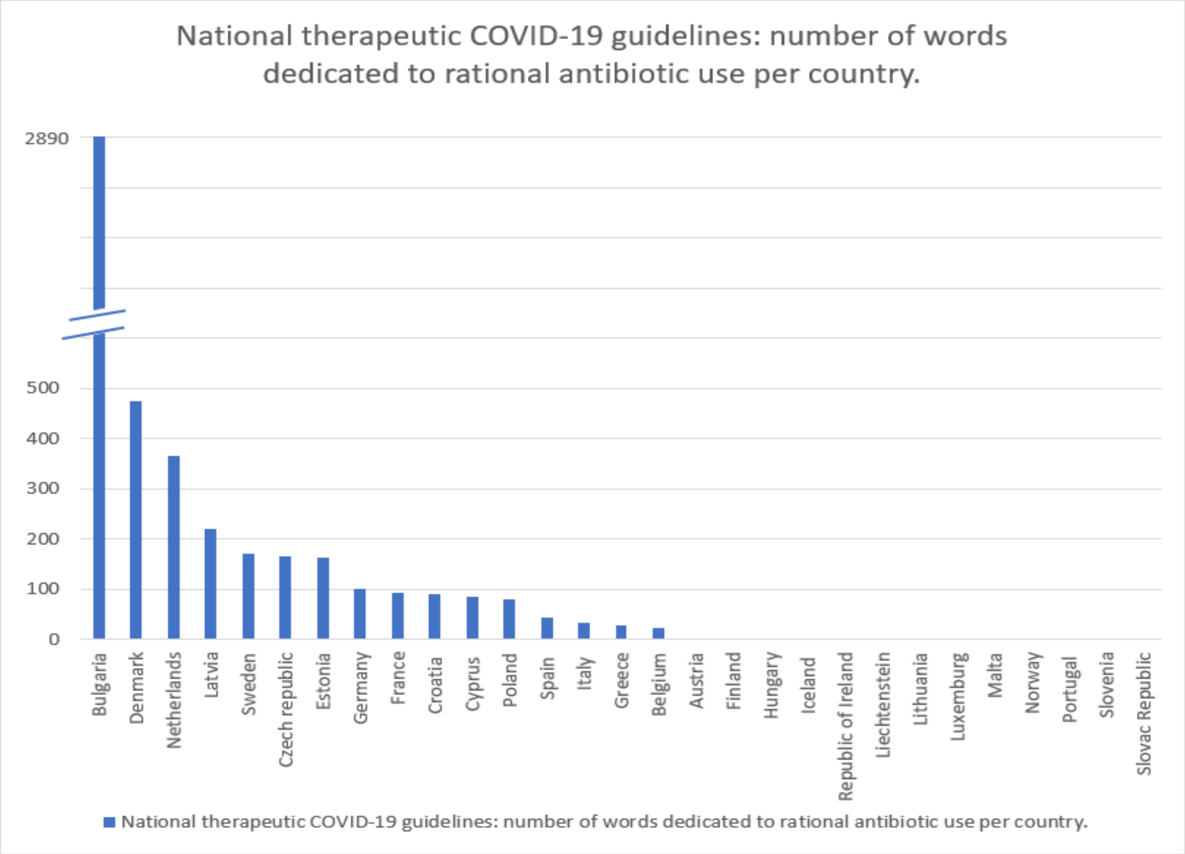

Created with Mapchart: https://www.mapchart.net/europe.html.
